# Supplementary material for: Impacts of pleiotropy and migration on repeated genetic adaptation
Source: Genetics. 2024 Jul 12;228(1):iyae111. doi: 10.1093/genetics/iyae111 (PMC11373517; doi:10.1093/genetics/iyae111)
Supplement: iyae111_Supplementary_Data [file iyae111_supplementary_data.zip › Figure_S1_GENETICS-2024-307073.pdf]

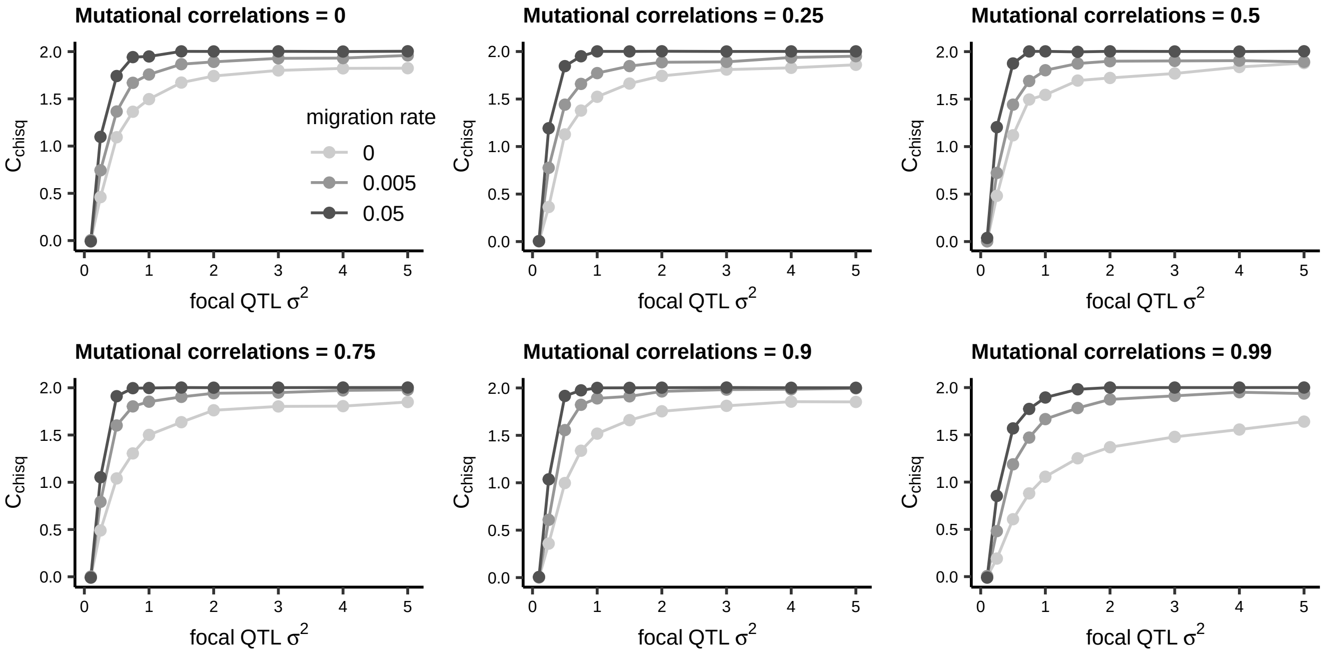

Mutational correlations = 0
Mutational correlations = 0.25
Mutational correlations = 0.5
Mutational correlations = 0.75
Mutational correlations = 0.9
Mutational correlations = 0.99
C_{chisq}
focal QTL σ^2
migration rate
0
0.005
0.05
