# Supplementary material for: Impacts of pleiotropy and migration on repeated genetic adaptation
Source: Genetics. 2024 Jul 12;228(1):iyae111. doi: 10.1093/genetics/iyae111 (PMC11373517; doi:10.1093/genetics/iyae111)
Supplement: iyae111_Supplementary_Data [file iyae111_supplementary_data.zip › Figure_S2_GENETICS-2024-307073.pdf]

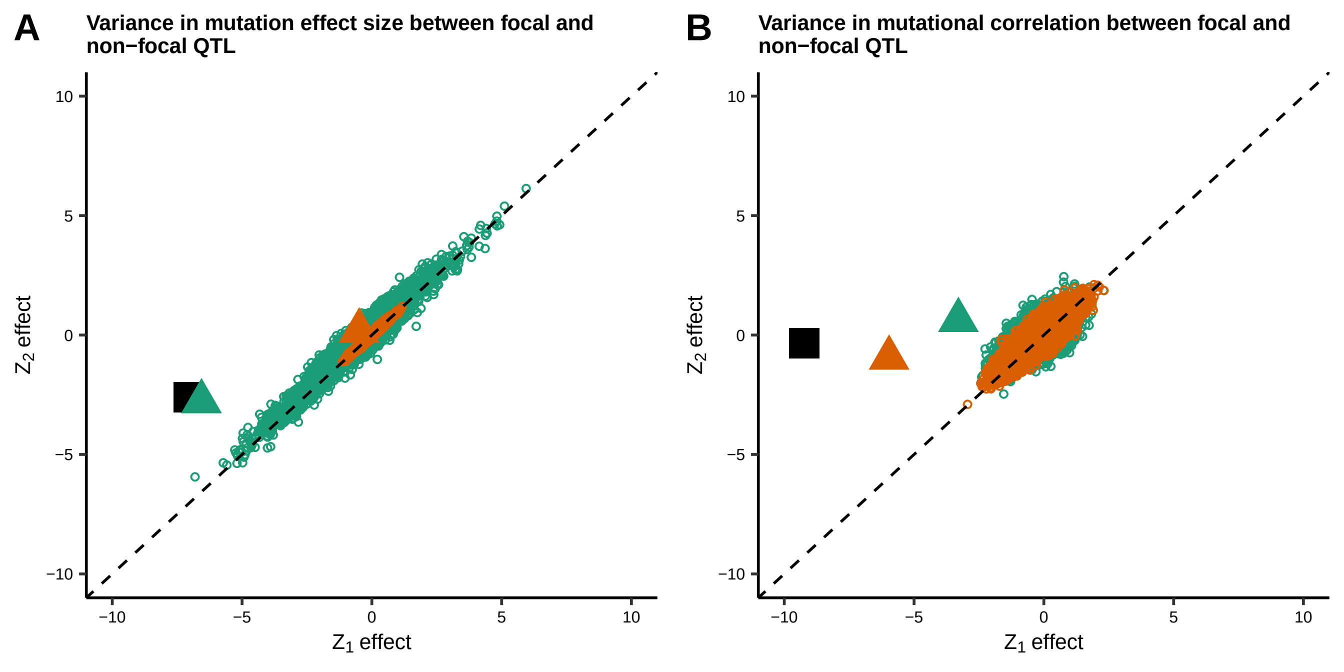
A
Variance in mutation effect size between focal and non−focal QTL
B
Variance in mutational correlation between focal and non−focal QTL
10
5
0
−5
−10
Z_2 effect
Z_1 effect
