# Supplementary material for: Impacts of pleiotropy and migration on repeated genetic adaptation
Source: Genetics. 2024 Jul 12;228(1):iyae111. doi: 10.1093/genetics/iyae111 (PMC11373517; doi:10.1093/genetics/iyae111)
Supplement: iyae111_Supplementary_Data [file iyae111_supplementary_data.zip › Figure_S6_GENETICS-2024-307073.pdf]

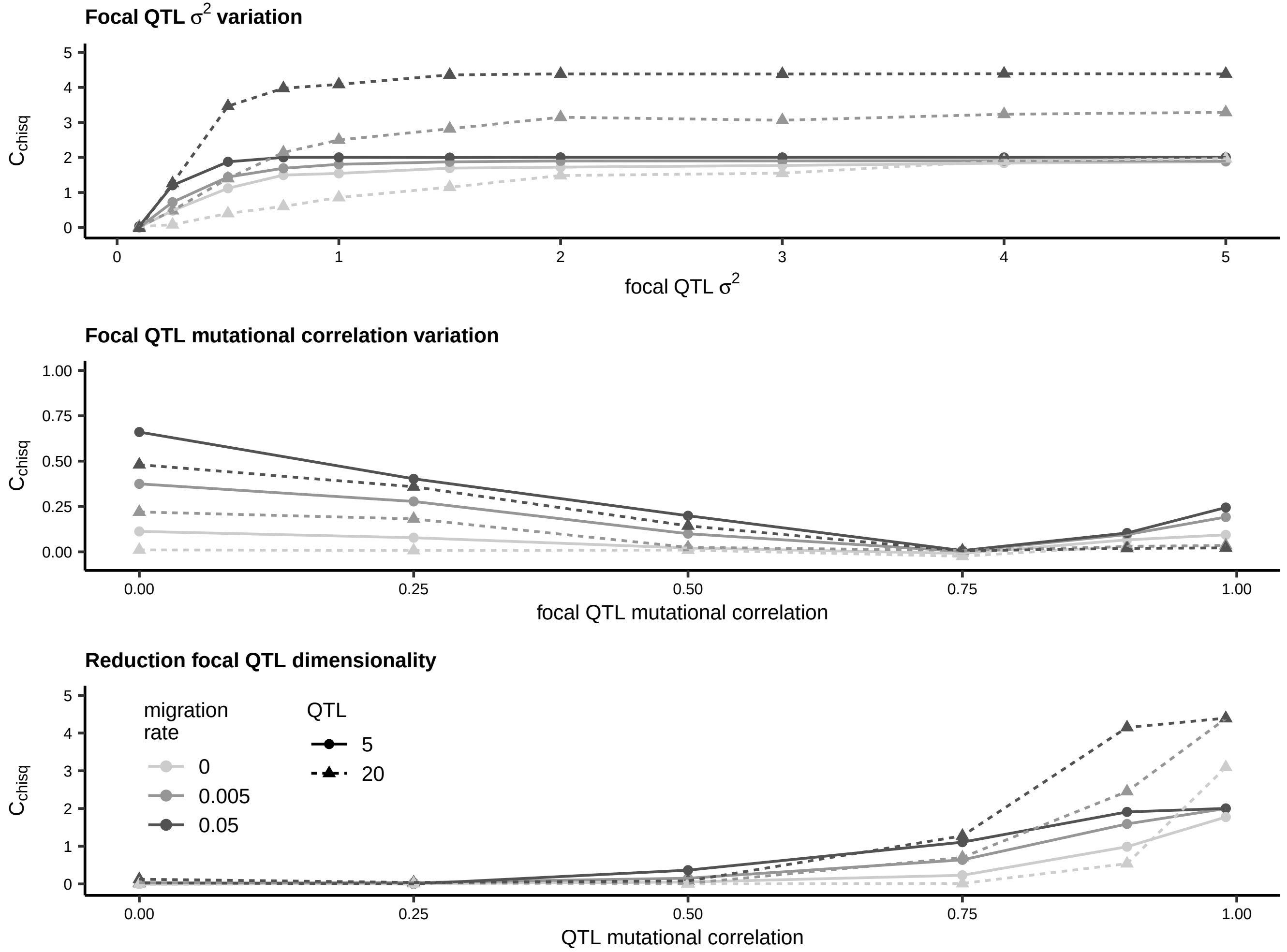
Focal QTL σ² variation
C_chisq
focal QTL σ²
Focal QTL mutational correlation variation
C_chisq
focal QTL mutational correlation
Reduction focal QTL dimensionality
C_chisq
migration rate
0
0.005
0.05
QTL
5
20
QTL mutational correlation
