# Supplementary material for: Impacts of pleiotropy and migration on repeated genetic adaptation
Source: Genetics. 2024 Jul 12;228(1):iyae111. doi: 10.1093/genetics/iyae111 (PMC11373517; doi:10.1093/genetics/iyae111)
Supplement: iyae111_Supplementary_Data [file iyae111_supplementary_data.zip › Supplementary_Figure_Legends_GENETICS-2024-307073.docx]

**Supplementary figure legends**

**Figure S1.** Repeatability (*C_chisq_*) in *Z_1_* against focal QTL *σ^2^* where the *σ^2^* for non-focal QTL is 0.1. These simulations use deleterious pleiotropy, two phenotypes (one divergent and one non-divergent). Simulations were run for 20,000 generations and 1000 replicates, with statistics calculated across replicates.

**Figure S2.** The mutations driving adaptation when one phenotype is under spatially divergent selection and the other is under spatially uniform selection (deleterious pleiotropy). Points show phenotypic effects for mutations segregating in *d_2_* across 1000 replicates for two example combinations of parameters (variance in mutation effect size, A: focal QTL *σ^2^* = 5; non-focal QTL *σ^2^* = 0.1; mutational correlations = 0.99 at all QTL; variance in mutational correlation, B: *σ^2^* = 0.5 for all QTL; focal QTL mutational correlations = 0.75; non-focal QTL mutational correlations = 0.9). Green points represent mutations at the focal QTL; orange points represent mutations at non-focal QTL. The mean divergence across replicates for focal and non-focal QTL is represented by green and orange triangles respectively, and the mean overall divergence by the black square. In both panels there are five QTL and migration rate = 0.005.

**Figure S3.** Divergence in *Z_1_* and repeatability (*C_chisq_*) over generations for four relationships between demes: where deme *d_2_* shifts to a new optimum in both traits *Z_1_* and *Z_2_* (divergent selection for both traits) without (A) and with (B) migration between *d_1_* and *d_2_*, and where *d_2_* shifts to new optimum in only trait *Z_1_* (divergent selection for one trait dimension and uniform selection for the other) without (C) and with (D) migration between *d_1_* and *d_2_*. *σ^2^* is 0.5 for the focal QTL and 0.1 for non-focal QTL. Mutational correlations are uniform at all QTL. These simulations use two phenotypes (one with divergent optima and the other with one non-divergent optima), and were run for 200,000 generations and 1000 replicates, with statistics calculated across replicates.

**Figure S4.** Repeatability (*C_chisq_*) in *Z_1_* against focal QTL mutational correlation for varying values of non-focal QTL mutation correlation (top row), the mean proportion of all *GV* explained by *GV* at the focal QTL (second row), and divergence between demes in *Z_1_* (third row) and *Z_2_* (bottom row). These simulations use deleterious pleiotropy an *σ^2^* of 0.1 and two phenotypes (one divergent and one non-divergent). They were run for 20,000 generations and 1000 replicates, with statistics calculated across replicates.

**Figure S5.** The effects of increasing trait dimensionality on repeatability (*C_chisq_*) arising from variation in *σ^2^* at the focal QTL where deme *d_2_* shifts to a new optimum in both trait dimensions *Z_1_* and *Z_2_* (divergent selection for both traits) with and without migration between *d_1_* and *d_2_* (A), and where *d_2_* shifts to new optimum in only *Z_1_* (divergent selection for one trait and uniform selection for the other) with and without migration between *d_1_* and *d_2_* (B). *σ^2^* at the focal QTL is varied, *σ^2^* for non-focal QTL is 0.1 and mutational correlations at all QTL are 0. Simulations were run for 20,000 generations and 1000 replicates, with statistics calculated across replicates.

**Figure S6.** Effects of increasing the number of QTL modelled from five (solid lines, circle points) to 20 (dashed lines, triangle points). In the top pane we examine effect-magnitude variation at the focal QTL (as in Fig. 2), with mutational correlations for all QTL fixed at 0.5. In the middle pane we examine mutational correlation variation at the focal QTL (as in Fig. 3), with mutational correlations at non-focal QTL of 0.75 and *σ^2^* at 0.5. In the lower pane we examine a reduction in dimensionality at the focal QTL (as in Fig. 5), where the total number of phenotypes is two and *σ^2^* is 0.5. These simulations use deleterious pleiotropy and two phenotypes (one divergent and one non-divergent). They were run for 20,000 generations and 1000 replicates, with statistics calculated across replicates.
