# Supplementary figures and images for: Impacts of pleiotropy and migration on repeated genetic adaptation
Source: Genetics. 2024 Jul 12;228(1):iyae111. doi: 10.1093/genetics/iyae111 (PMC11373517; doi:10.1093/genetics/iyae111)

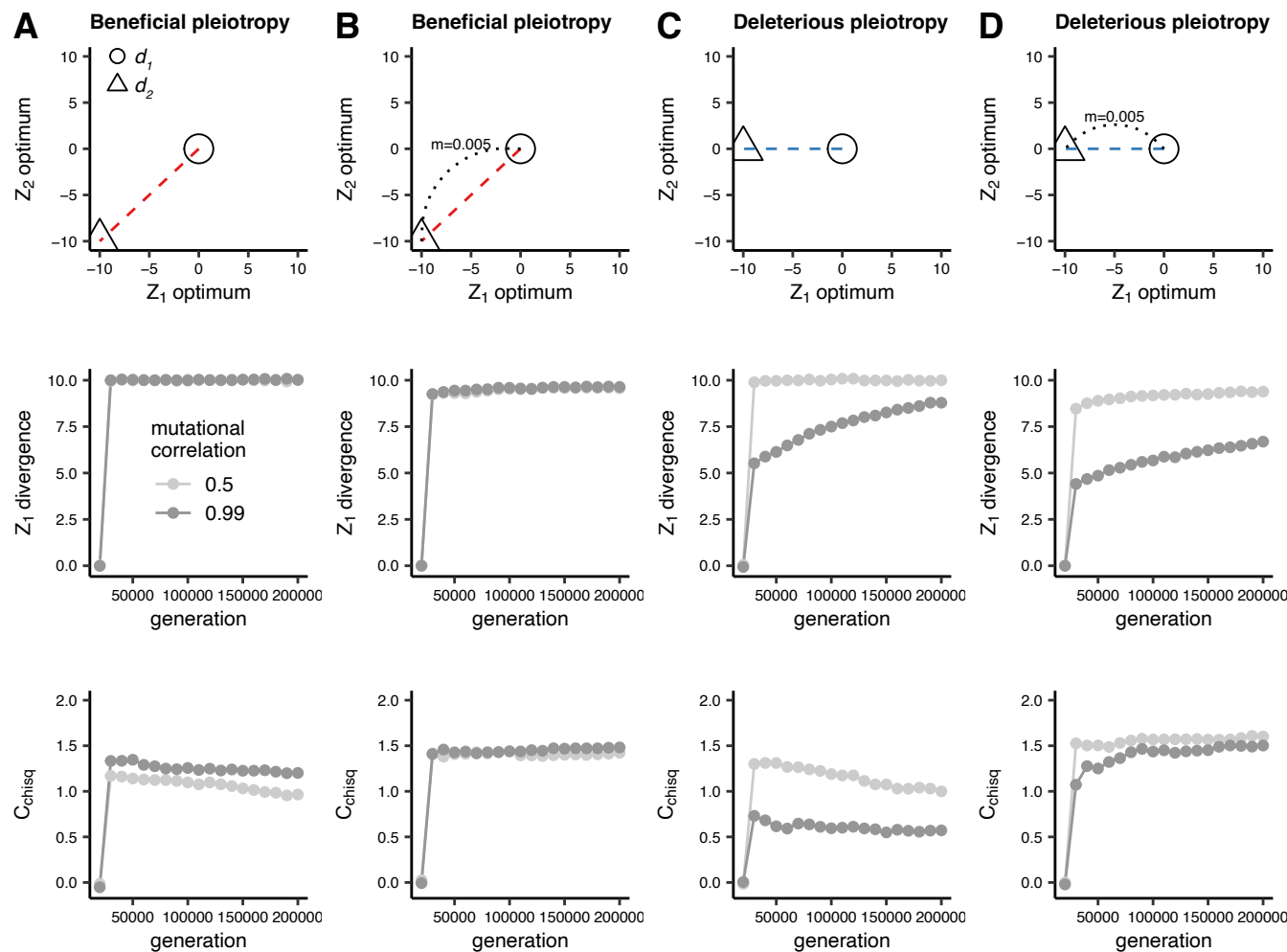

Supplement: iyae111_Supplementary_Data [file iyae111_supplementary_data.zip › Figure_S3_GENETICS-2024-307073.pdf]

Migration rate = 0

Migration rate = 0.005

Migration rate = 0.05

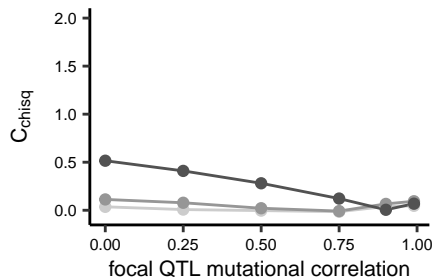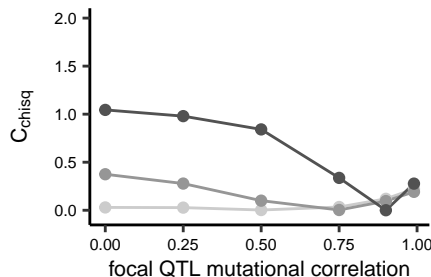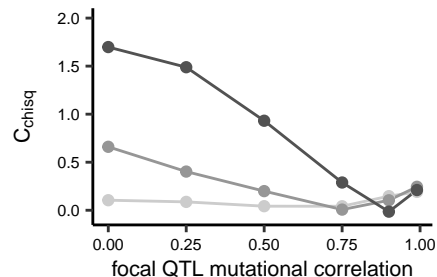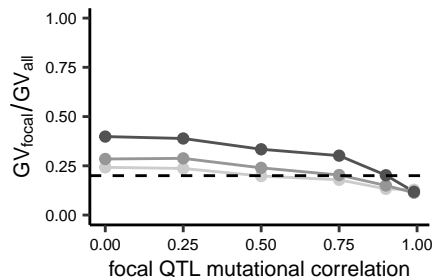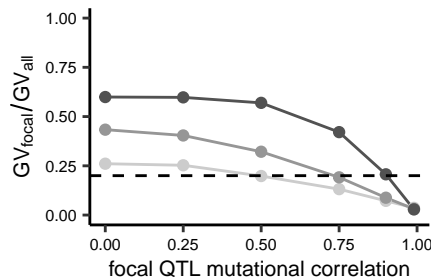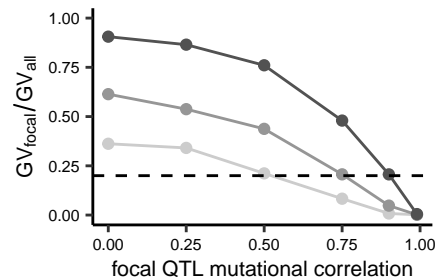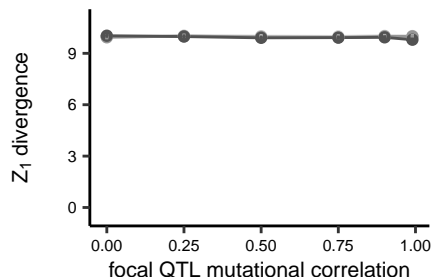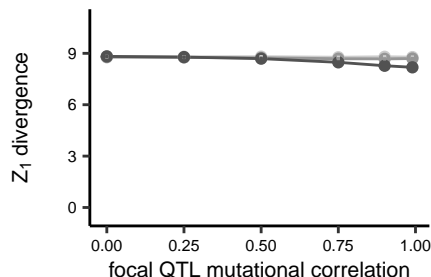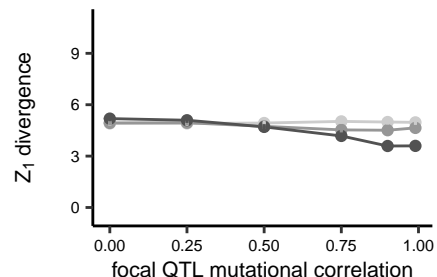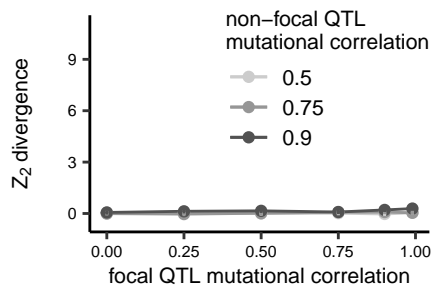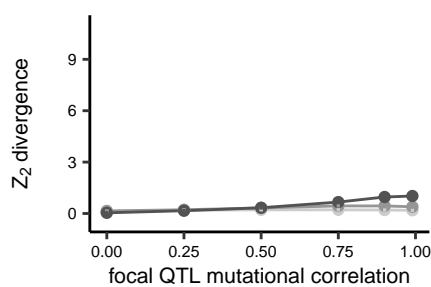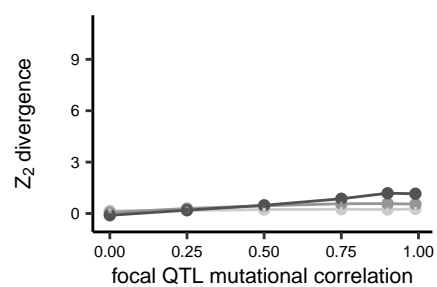

Supplement: iyae111_Supplementary_Data [file iyae111_supplementary_data.zip › Figure_S4_GENETICS-2024-307073.pdf]

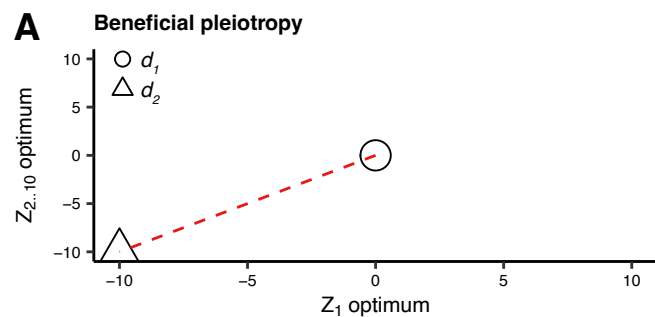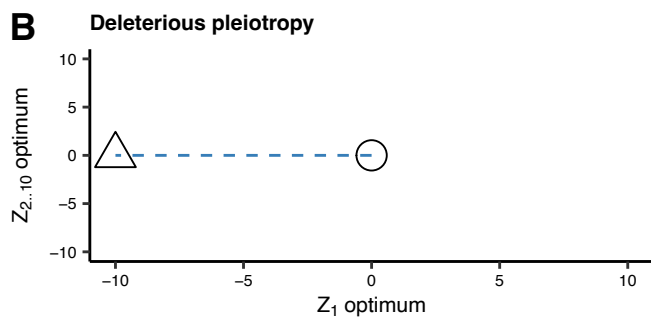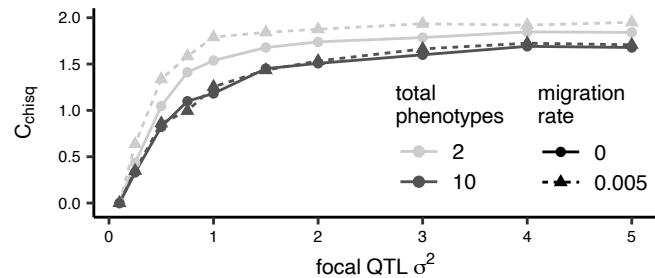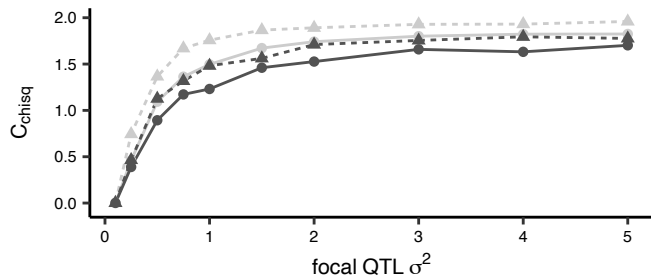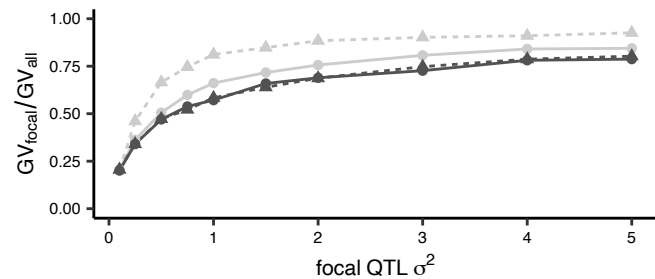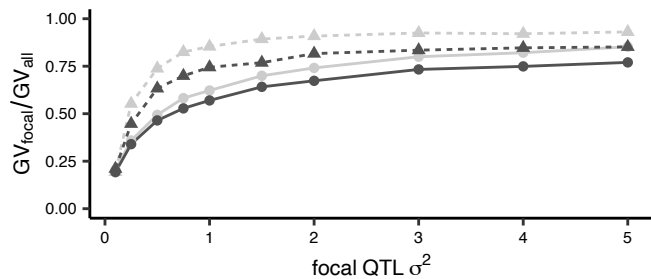

Supplement: iyae111_Supplementary_Data [file iyae111_supplementary_data.zip › Figure_S5_GENETICS-2024-307073.pdf]
